# Postherpetic Neuralgia Mimicking Lumbar Radiculopathy in the Same Dermatome: A Diagnostic Challenge

**DOI:** 10.4274/TJAR.2026.252377

**Published:** 2026-06-26

**Authors:** Onurcan Balık, Sefa Tan, Sema Tuncer Uzun, Ruhiye Reisli

**Affiliations:** 1 Necmettin Erbakan University Faculty of Medicine Hospital Department of Pain Medicine, Konya, Türkiye

**Keywords:** Dorsal root ganglion, pain, postherpetic neuralgia, pulsed radiofrequency

Dear Editor,

Postherpetic neuralgia (PHN) is a well-recognized and often debilitating complication of herpes zoster, characterized by persistent neuropathic pain after resolution of cutaneous lesions.^1, 2^ Although thoracic dermatomes are most commonly involved, lumbosacral herpes zoster is relatively rare and may easily be misinterpreted as lumbar radiculopathy, particularly during the prodromal phase when neuropathic pain precedes the appearance of skin lesions.^3, 4^

The coexistence of herpes zoster-related dorsal root ganglion (DRG) irritation and structural nerve root compression within the same dermatome creates a unique diagnostic dilemma. Previous reports have described patients in whom herpes zoster–related sciatic pain closely mimicked lumbar disc herniation or spinal canal stenosis, resulting in misdiagnosis and delayed targeted treatment.^5, 6^

The patient presented with persistent low back pain and left gluteal pain radiating along the L5 dermatome. Two weeks before presenting to our clinic, vesicular lesions developed in the same dermatome, and he was diagnosed with herpes zoster. Lumbar magnetic resonance imaging demonstrated a concomitant L5-S1 disc herniation compressing the left L5 nerve root. Prominent neuropathic pain features, including a burning quality and allodynia, suggested viral involvement, whereas imaging findings supported a concomitant mechanical component, resulting in diagnostic uncertainty.

We therefore applied a combined interventional approach, consisting of pulsed radiofrequency (PRF) to the L5 DRG, followed by a transforaminal epidural steroid injection, to address both virus-induced neuronal hyperexcitability and inflammatory radicular compression (Figure 1). DRG-targeted interventions play a central role in PHN due to the involvement of sensory neurons in the initiation and maintenance of neuropathic pain.^7^ In addition, systematic reviews indicate that early nerve block and PRF applications may reduce the incidence and severity of PHN in patients with acute herpes zoster.^8^

Following the combined intervention, the patient’s numeric rating scale score decreased from 7 to 1 and the leeds assessment of neuropathic symptoms and signs score from 15 to 0 within the first hour, with sustained analgesic benefit at the three-week follow-up.

This experience underscores the importance of recognizing overlapping viral and mechanical pain mechanisms in patients presenting with atypical lumbosacral radicular pain. Early consideration of combined DRG-targeted interventions may not only provide rapid diagnostic clarification, but may also play a critical role in preventing progression to chronic PHN in complex clinical scenarios. In patients with prominent neuropathic pain features (e.g., burning pain and allodynia), herpes zoster should be considered even in the absence of rash (zoster sine herpete). In such cases, diagnostic tests (VZV DNA detection and VZV-specific IgM and IgG antibodies) may support the differential diagnosis.

## Figures and Tables

**Figure 1:**
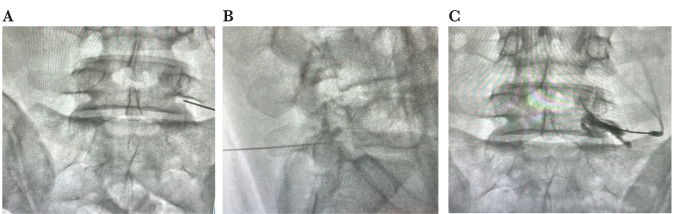
Left L5 transforaminal epidural steroid injection: A, AP view; B, Lateral view; C, AP view with contrast distribution. AP, anteroposterior.
